# Quality and reliability of short videos about type A aortic dissection on Bilibili and TikTok: a cross-sectional study

**DOI:** 10.3389/fdgth.2026.1964412

**Published:** 2026-10-21

**Authors:** Keyu Wei, Mengqi Liu, Yuji Yan, Ruiying Lan, Yizi Zhang, Xiaolei Han, Ying Wang, Wenxu He, Xinhua Tang, Naizhong Zhang, Xiaolin Gao, Chunyu Tian, Xiaoling Wang

**Affiliations:** 1 Qian’an Hospital of Traditional Chinese Medicine, Qian’an, China; 2 Hebei North University, Zhangjiakou, China; 3 Nanjing University of Chinese Medicine, Nanjing, China; 4 College of Traditional Chinese Medicine, Hebei North University, Zhangjiakou, China; 5 North China University of Science and Technology, Caofeidian New City, Tangshan, China

**Keywords:** Bilibili, GQS, mDISCERN, TikTok, type A aortic dissection, video quality

## Abstract

**Background:**

Type A aortic dissection (TAAD) is a life-threatening cardiovascular emergency. Short-video platforms such as TikTok and Bilibili have become important channels for health information dissemination, but the quality and reliability of TAAD-related videos remain unclear.

**Objective:**

This study aimed to evaluate the quality and reliability of TAAD-related short videos on TikTok and Bilibili, with particular attention to platform differences and uploader characteristics.

**Methods:**

The top 250 videos ranked by comprehensive ranking on each platform were screened using the Chinese keyword “A型主动脉夹层.” A total of 391 eligible videos were included. Uploaders were classified as medical practitioners, individual users, or institutions. Video quality and reliability were assessed using the Global Quality Score (GQS), modified DISCERN (mDISCERN), JAMA benchmarks, and Video Information Quality Instrument (VIQI). Multivariable regression analyses were performed to identify factors independently associated with video quality.

**Results:**

TikTok videos showed higher engagement, whereas Bilibili videos were longer and provided broader coverage of epidemiology, etiology, and clinical presentation-related content. TikTok remained independently associated with higher VIQI scores after adjustment. Medical practitioners and institutions generally achieved higher VIQI and JAMA scores than individual users, and content coverage score was independently associated with higher video quality. However, mDISCERN scores did not differ significantly among uploader types.

**Conclusion:**

TAAD-related short videos demonstrated moderate overall quality but limited reliability. Platform characteristics, uploader type, and content coverage were associated with assessed information quality. Professional participation may improve some aspects of health communication, but uploader identity alone does not ensure higher reliability or transparency.

## 1 Introduction

Type A aortic dissection (TAAD) is a catastrophic and life-threatening cardiovascular emergency characterized by a tear in the aortic wall layers, leading to the formation of a false lumen that rapidly propagates along the aorta (1). The condition is marked by sudden onset, high morbidity, and devastating mortality, with rates reaching 50%–68% within 48 h and up to 90% within three months if untreated (2). In China, the epidemiological profile of TAAD is particularly severe, with a higher prevalence of complex chronic cases and aneurysmal progression, a younger age of onset, and compounded by widespread hypertension with low awareness and control rates (3). Despite advances in imaging and surgical care, in-hospital mortality remains substantial, ranging from 15% to 30% (1). Given its acute and lethal nature, improving public recognition of TAAD symptoms and the urgency of seeking immediate medical care is critical for reducing mortality and alleviating the overall disease burden.

Over the past decade, social media platforms have emerged as primary sources of health information for the public in the digital health era (4, 5). Short-video platforms such as TikTok and Bilibili, with billions of active users globally, offer unique advantages for disseminating medical knowledge, including high accessibility, rapid sharing, and engaging audiovisual formats (4). Over 80% of internet users now seek health information online, increasingly preferring video over text (6, 7). However, the quality, reliability, and transparency of such content remain important concerns, as social media platforms allow information to be uploaded by heterogeneous sources with varying levels of professional oversight. Prior studies evaluating short videos on thyroid cancer (8), diabetic kidney disease (9), uraemia (10), and inflammatory bowel diseases (11) have consistently identified significant deficiencies in information quality and reliability. Despite the life-threatening nature of TAAD, the quality, reliability, and completeness of TAAD-related short videos have not been adequately characterized, raising concerns about whether currently available content provides sufficiently comprehensive and transparent health information.

This study aims to assess the quality and reliability of TAAD-related short videos on Bilibili and TikTok using validated instruments including the Global Quality Score (GQS), modified DISCERN (mDISCERN), JAMA benchmarks, and the Video Information Quality Instrument (VIQI). The findings will provide evidence-based insights for healthcare professionals, platform operators, and policymakers to optimize the digital health information ecosystem and enhance public cardiovascular health literacy.

## 2 Materials and methods

### 2.1 Ethical considerations

All data were derived from publicly accessible videos on Bilibili and TikTok platforms. No personal privacy information was involved. This study was exempted from ethical review as it involved analysis of publicly available data only.

### 2.2 Search strategy and data collection

This descriptive cross-sectional study was conducted in Jiangsu, China (Figure 1). Data collection was performed on August 2, 2026, using the web versions of TikTok and Bilibili on the same computer and under the same network environment. Both platform searches were completed on the same day by the same investigator using newly created, logged-in accounts with no prior search, viewing, following, liking, commenting, or other interaction history. Before data collection, the browser cache was cleared and location services were disabled to minimize the potential influence of previous browsing activity and location-based personalization.

**Figure 1:**
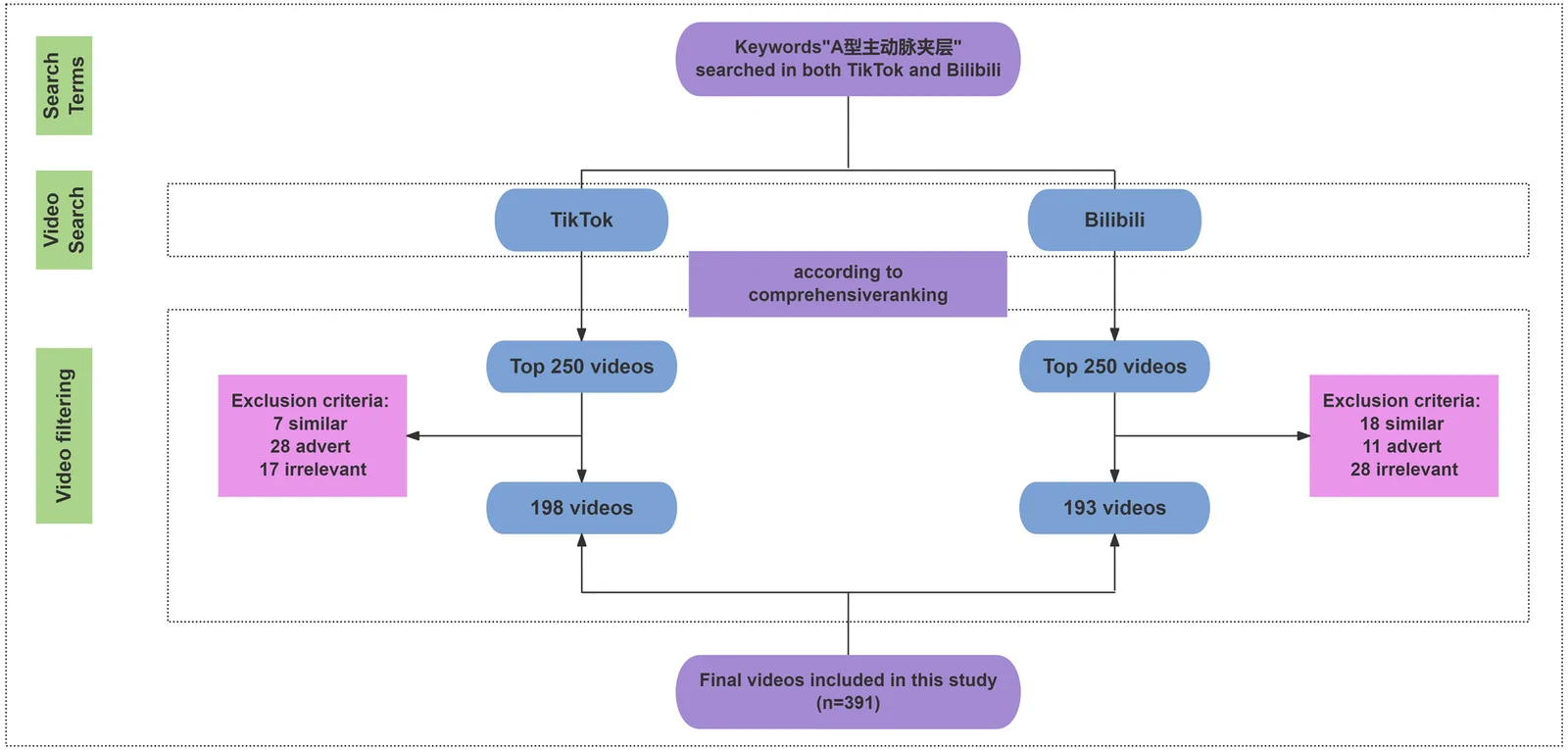
Search strategy and video filtering program.

To gather data, a systematic search was conducted on both platforms using the Chinese search term “A型主动脉夹层” (Type A aortic dissection). This disease-specific term was selected to improve retrieval specificity for Stanford type A aortic dissection and to reduce the inclusion of videos primarily concerning other types of aortic dissection. To ensure data stability and minimize potential bias from short-term fluctuations in viewer engagement, videos published within seven days before data collection were excluded.

Videos were sorted using each platform's default “comprehensive ranking” function. This mechanism integrates multiple factors, including video completion rate (e.g., viewers watching for more than 5 s), like rate, comment rate, follow rate, and upload time, to rank videos according to recency and engagement (12). The top 250 videos from each platform were screened. This fixed sampling frame was used to capture a broad set of highly ranked videos that users would be relatively likely to encounter under the default search setting, while ensuring an equivalent retrieval depth across the two platforms and maintaining the feasibility of manual screening and detailed video assessment. The top-250 cutoff was intended to characterize highly visible search results rather than to provide an exhaustive representation of all TAAD-related videos available on either platform.

From the initially retrieved videos, a subsequent screening process was applied based on predefined exclusion criteria. Exclusion criteria included: (1) videos unrelated to TAAD; (2) duplicate videos; (3) advertisement videos; and (4) videos entirely devoid of visual content. This screening process yielded the final set of eligible videos for analysis. For each included video, the following information was recorded: video title, platform, upload date, video duration, number of likes, collections, comments, shares, and uploader type. Video quality and reliability were subsequently assessed.

### 2.3 Video classification

Uploaders were categorized into three groups: (1) Medical practitioners (certified healthcare professionals); (2) Individual users (non-medical professionals, self-media accounts); (3) Institutions (hospitals, medical organizations, official media).

### 2.4 Quality assessment

Video quality was assessed using four validated instruments:

#### 2.4.1 Global quality score (GQS)

A 5-point scale assessing video quality based on comprehensiveness, usability, and overall presentation quality (1 = very poor, 5 = excellent) (13, 14).

#### 2.4.2 Modified DISCERN tool (mDISCERN)

A 5-point scale evaluating reliability across five parameters: clarity, relevance, traceability, robustness, and fairness (15, 16).

#### 2.4.3 JAMA benchmarks

A 4-point scale assessing authorship attribution, disclosure, currency, and peer-review status (17).

#### 2.4.4 Video information quality instrument (VIQI)

A 20-point scale evaluating video information quality across multiple dimensions (16, 18).

All 391 eligible videos were independently evaluated by two medical students. Before formal assessment, both raters received standardized training on the scoring criteria and application of the GQS, mDISCERN, JAMA benchmarks, and VIQI instruments. During the formal assessment, the two raters evaluated each video independently and had no access to each other's ratings. Blinding to platform and uploader identity was not performed because platform-specific features and uploader information could be visible in the original videos. For subsequent analyses, the final score for each video was calculated as the mean of the two independent ratings.

### 2.5 Statistical analysis

Statistical analyses were performed using WPS, GraphPad Prism, and Zstats software. Inter-rater reliability was assessed using quadratic weighted Cohen's kappa for the ordinal GQS, mDISCERN, and JAMA scores. For the VIQI total score, inter-rater reliability was assessed using an intraclass correlation coefficient (ICC) based on a two-way random-effects, absolute-agreement, single-measure model. Reliability analyses were based on the two raters' original independent scores before averaging.

The Shapiro–Wilk test was used to assess the normality of continuous variables. Data that were not normally distributed are presented as medians with interquartile ranges (IQRs). Group comparisons were conducted using the Mann–Whitney *U*-test for two groups and the Kruskal–Wallis test for three or more groups. Multivariable regression analyses were performed to identify independent factors associated with video quality scores. Robust linear regression was used for the VIQI total score, whereas ordinal logistic regression models were applied for GQS, mDISCERN, and JAMA scores. Candidate predictors included platform, uploader type, log-transformed video duration, and TAAD-related content coverage score. The Benjamini-Hochberg false discovery rate (FDR) correction was applied separately within each regression model to account for multiple comparisons. Other comparative and correlation analyses were considered exploratory and interpreted with caution. Spearman's correlation analysis was used to evaluate relationships between video characteristics and quality scores. A *P* value < 0.05 was considered statistically significant.

## 3 Results

### 3.1 Inter-rater reliability

Inter-rater reliability analysis demonstrated high agreement between the two independent raters across all assessment instruments. The quadratic weighted Cohen's kappa values were 0.894 for GQS, 0.892 for mDISCERN, and 0.937 for JAMA. The ICC for the VIQI total score was 0.965, indicating high agreement between raters.

### 3.2 Video characteristics

A total of 391 videos were included in this study, comprising 193 videos from Bilibili and 198 videos from TikTok (Table 1). The search and filtering process is shown in Figure 1. The median video length was 107.0 s (Q_1_, Q_3_: 49.0, 273.5). Regarding audience engagement, the videos received a median of 129.0 likes (Q_1_, Q_3_: 28.0, 542.0), 42.0 collections (Q_1_, Q_3_: 6.0, 193.0), 18.0 comments (Q_1_, Q_3_: 2.0, 88.5), and 17.0 shares (Q_1_, Q_3_: 3.0, 89.0). In terms of video content, treatment was the most frequently covered topic, appearing in 295 videos (75.4%), followed by diagnosis in 262 videos (67.0%), etiology in 190 videos (48.6%), clinical presentation in 181 videos (46.3%), prognosis in 155 videos (39.6%), and epidemiology in 111 videos (28.4%). Prevention was the least frequently covered topic, appearing in only 61 videos (15.6%). Regarding video sources, medical practitioners contributed the largest proportion of videos (227/391, 58.1%), followed by individual users (136/391, 34.8%) and institutions (28/391, 7.2%). For video quality and reliability, the median VIQI score was 13.0 (Q_1_, Q_3_: 11.0, 14.0), while the median GQS score was 3.0 (Q_1_, Q_3_: 3.0, 4.0), suggesting moderate overall quality. The median mDISCERN score was 2.0 (Q_1_, Q_3_: 2.0, 2.0), and the median JAMA score was 2.0 (Q_1_, Q_3_: 2.0, 2.0), indicating that the reliability and transparency of the videos remained limited.

**Table 1:** General characteristics, quality, and reliability of the videos.

Variables	Total (*n* = 391)
General information	
Video length(s), M (Q_1_, Q_3_)	107.0 (49.0, 273.5)
Likes, M (Q_1_, Q_3_)	129.0 (28.0, 542.0)
Collections, M (Q_1_, Q_3_)	42.0 (6.0, 193.0)
Comments, M (Q_1_, Q_3_)	18.0 (2.0, 88.5)
Shares, M (Q_1_, Q_3_)	17.0 (3.0, 89.0)
Video content	
Epidemiology, *n* (%)	111.0 (28.4)
Etiology, *n* (%)	190.0 (48.6)
Clinical Presentation, *n* (%)	181.0 (46.3)
Diagnosis, *n* (%)	262.0 (67.0)
Treatment, *n* (%)	295.0 (75.4)
Prevention, *n* (%)	61.0 (15.6)
Prognosis, *n* (%)	155.0 (39.6)
Video sources	
Medical practitioners, *n* (%)	227.0 (58.1)
Individual Users, *n* (%)	136.0 (34.8)
Institutions, *n* (%)	28.0 (7.2)
Video quality	
VIQI score, M (Q_1_, Q_3_)	13.0 (11.0, 14.0)
GQS score, M (Q_1_, Q_3_)	3.0 (3.0, 4.0)
mDISCERN score, M (Q_1_, Q_3_)	2.0 (2.0, 2.0)
JAMA score, M (Q_1_, Q_3_)	2.0 (2.0, 2.0)

M: Median, Q_1_: 1st Quartile, Q_3_: 3rd Quartile.

VIQI, Video Information Quality Instrument; GQS, Global Quality Score; mDISCERN, modified DISCERN tool; JAMA, JAMA Benchmarks.

### 3.3 Platform comparison

The distribution of videos between the two platforms was relatively balanced, with Bilibili accounting for 49.4% and TikTok accounting for 50.6% of all included videos (Figure 2A). However, significant differences were observed in several video characteristics between the two platforms (Table 2).

**Figure 2:**
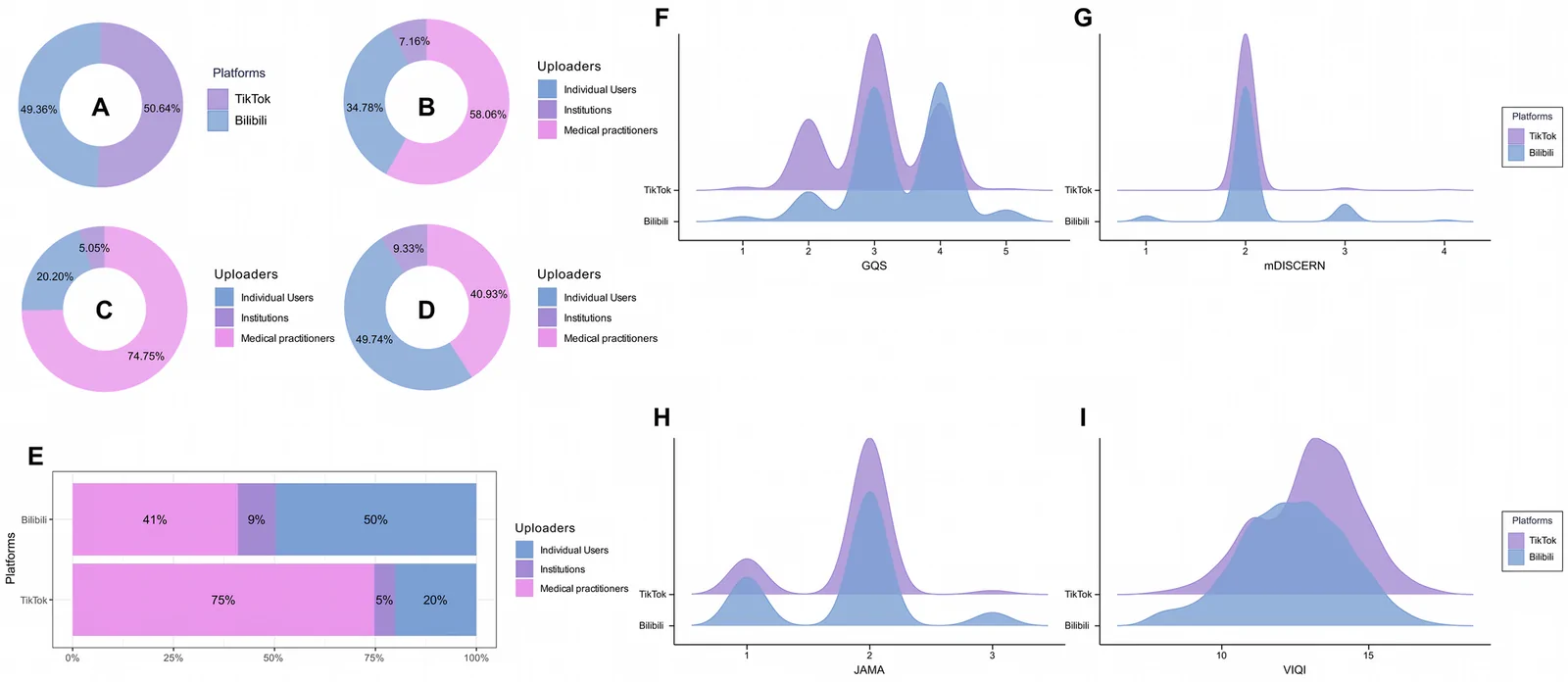
Distribution of video platforms, uploader types, and quality scores. **(A)** The proportion of videos from TikTok and Bilibili platforms; **(B)** Overall distribution of video uploaders among all included videos; **(C)** Distribution of video uploaders on TikTok; **(D)** Distribution of video uploaders on Bilibili; **(E)** Comparison of uploader composition between TikTok and Bilibili platforms; (**F-I**): Distribution of GQS/mDISCERN/JAMA/VIQI scores of videos between TikTok and Bilibili platforms.

**Table 2:** General information, quality, and reliability scores of TAAD videos on TikTok and BiliBili.

Variables	Total (*n* = 391)	Bilibili (*n* = 193)	TikTok (*n* = 198)	*P*	FDR-adjusted *P*
General information					
Video length(s), M (Q_1_, Q_3_)	107.0 (49.0, 273.5)	213.0 (86.0, 668.0)	77.0 (40.0, 143.0)	**<0**.**001**	**–**
Likes, M (Q_1_, Q_3_)	129.0 (28.0, 542.0)	33.0 (7.0, 303.0)	245.5 (95.5, 812.5)	**<0**.**001**	**–**
Collections, M (Q_1_, Q_3_)	42.0 (6.0, 193.0)	49.0 (6.0, 179.0)	37.5 (6.3, 215.0)	0.958	**–**
Comments, M (Q_1_, Q_3_)	18.0 (2.0, 88.5)	4.0 (0.0, 38.0)	51.0 (13.0, 180.8)	**<0**.**001**	**–**
Shares, M (Q_1_, Q_3_)	17.0 (3.0, 89.0)	14.0 (2.0, 59.0)	24.0 (5.0, 160.3)	**0**.**004**	**–**
Video content					
Epidemiology, *n* (%)	111.0 (28.4)	67.0 (34.7)	44.0 (22.2)	**0**.**009**	**0.020**
Etiology, *n* (%)	190.0 (48.6)	111.0 (57.5)	79.0 (39.9)	**<0**.**001**	**0.003**
Clinical Presentation, *n* (%)	181.0 (46.3)	108.0 (56.0)	73.0 (36.9)	**<0**.**001**	**0.002**
Diagnosis, *n* (%)	262.0 (67.0)	139.0 (72.0)	123.0 (62.1)	0.056	0.079
Treatment, *n* (%)	295.0 (75.4)	143.0 (74.1)	152.0 (76.8)	0.619	0.654
Prevention, *n* (%)	61.0 (15.6)	28.0 (14.5)	33.0 (16.7)	0.654	0.654
Prognosis, *n* (%)	155.0 (39.6)	66.0 (34.2)	89.0 (44.9)	**0**.**038**	0.067
Video sources					
Medical practitioners, *n* (%)	227.0 (58.1)	79.0 (40.9)	148.0 (74.7)		**–**
Individual Users, *n* (%)	136.0 (34.8)	96.0 (49.7)	40.0 (20.2)		**–**
Institutions, *n* (%)	28.0 (7.2)	18.0 (9.3)	10.0 (5.1)		**–**
Video quality					
VIQI score, M (Q_1_, Q_3_)	13.0 (11.0, 14.0)	12.0 (11.0, 14.0)	13.0 (12.0, 14.0)	**<0**.**001**	**–**
GQS score, M (Q_1_, Q_3_)	3.0 (3.0, 4.0)	3.0 (3.0, 4.0)	3.0 (3.0, 4.0)	**<0**.**001**	**–**
mDISCERN score, M (Q_1_, Q_3_)	2.0 (2.0, 2.0)	2.0 (2.0, 2.0)	2.0 (2.0, 2.0)	**0**.**027**	**–**
JAMA score, M (Q_1_, Q_3_)	2.0 (2.0, 2.0)	2.0 (2.0, 2.0)	2.0 (2.0, 2.0)	0.546	**–**

M: Median, Q_1_: 1st Quartile, Q_3_: 3rd Quartile.

Continuous variables were compared using the Mann–Whitney *U*-test. Categorical content coverage variables were compared using the chi-square test or Fisher's exact test, as appropriate. Because seven content domains were evaluated, the Benjamini-Hochberg false discovery rate (FDR) correction was applied to content coverage comparisons.

Bilibili videos were significantly longer than TikTok videos, with a median length of 213.0 s (Q_1_, Q_3_: 86.0, 668.0) compared with 77.0 s (Q_1_, Q_3_: 40.0, 143.0) on TikTok (*P* < 0.001). In contrast, TikTok videos showed significantly higher audience engagement. The median number of likes was 245.5 on TikTok and 33.0 on Bilibili (*P* < 0.001). TikTok videos also received more comments [51.0 (13.0, 180.8) vs. 4.0 (0.0, 38.0), *P* < 0.001] and shares [24.0 (5.0, 160.3) vs. 14.0 (2.0, 59.0), *P* = 0.004]. No significant difference was observed in collections between the two platforms (*P* = 0.958).

The composition of uploaders differed between platforms. On TikTok, medical practitioners accounted for the majority of videos (148/198, 74.7%), whereas individual users accounted for only 20.2%. In contrast, on Bilibili, individual users represented the largest uploader group (96/193, 49.7%), followed by medical practitioners (79/193, 40.9%) and institutions (18/193, 9.3%) (Figures 2C–E).

Regarding content coverage, treatment was the most frequently covered topic on both platforms. After FDR correction, Bilibili videos showed significantly higher coverage of epidemiology, etiology, and clinical presentation compared with TikTok videos. No significant differences were observed in diagnosis, treatment, prevention, or prognosis coverage between platforms. Prevention was insufficiently covered on both platforms (Table 2, Figure 3A).

**Figure 3:**
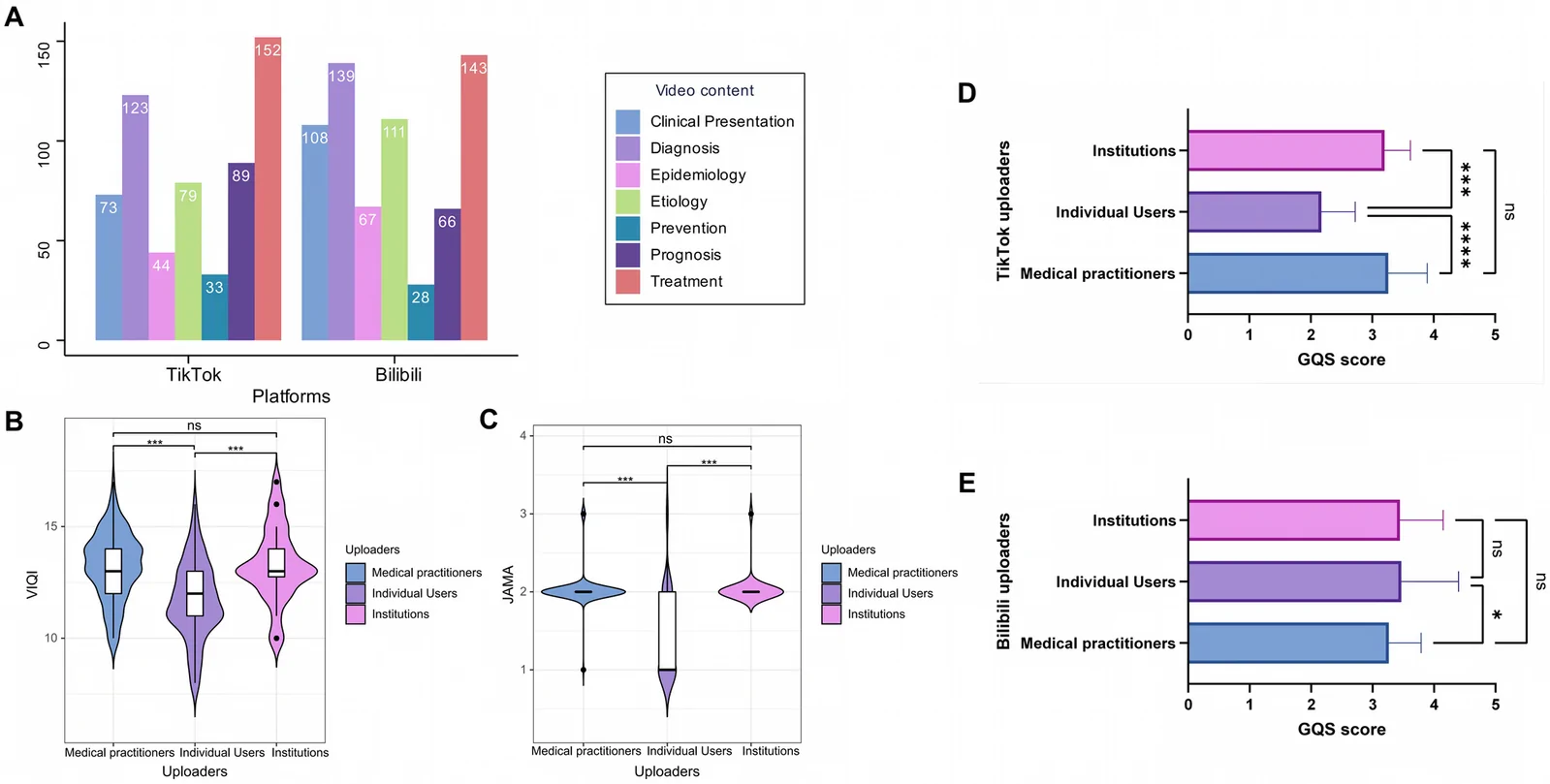
Comparison of video content coverage and quality scores among different platforms and uploader types. **(A)** The coverage of video content topics between TikTok and Bilibili platforms; **(B)** Distribution of VIQI scores across different uploader types; **(C)** Distribution of JAMA scores across different uploader types; **(D)** Comparison of GQS scores among different uploader types on TikTok; **(E)**: Comparison of GQS scores among different uploader types on Bilibili. (ns: *P* ≥ 0.05; *P < 0.05; ***P < 0.001).

For video quality assessment, TikTok videos had a higher median VIQI score than Bilibili videos [13.0 (12.0, 14.0) vs. 12.0 (11.0, 14.0), *P* < 0.001]. Although statistically significant differences were observed in GQS and mDISCERN scores between platforms, the median values and interquartile ranges were identical, suggesting that the magnitude and practical relevance of these differences may be limited and should therefore be interpreted cautiously. No significant difference was found in JAMA scores between Bilibili and TikTok (*P* = 0.546) (Figure 2).

### 3.4 Uploader characteristics

Among all included videos, 227 were uploaded by medical practitioners, 136 by individual users, and 28 by institutions. Significant differences were observed among uploader categories in video length, engagement indicators, VIQI score, and JAMA score (Table 3).

**Table 3:** Characteristics, quality, and reliability of TAAD videos by different uploaders on TikTok and Bilibili.

Variables	Total (*n* = 391)	Individual Users (*n* = 136)	Institutions (*n* = 28)	Medical practitioners (*n* = 227)	*P*
General information					
Video length(s), M (Q_1_, Q_3_)	107.0 (49.0, 273.5)	192.50 (76.8,720.8)	117.5 (83.8,209.0)	83.0 (42.0,203.5)	**<0**.**001**
Likes, M (Q₁, Q_3_)	129.0 (28.0, 542.0)	36.5 (10.0,131.0)	125.5 (33.5,1305.5)	278.0 (67.0,836.5)	**<0**.**001**
Collections, M (Q_1_, Q_3_)	42.0 (6.0, 193.0)	24.5 (5.0,105.8)	62.0 (20.3,258.8)	53.0 (8.0,231.0)	**0**.**009**
Comments, M (Q_1_, Q_3_)	18.0 (2.0, 88.5)	5.0 (1.0,44.5)	11.5 (1.8,107.3)	30.0 (6.5,125.5)	**<0**.**001**
Shares, M (Q_1_, Q_3_)	17.0 (3.0, 89.0)	8.0 (2.0,31.0)	21.5 (7.8,207.5)	28.0 (3.5,140.0)	**<0**.**001**
Video content					
Epidemiology, *n* (%)	111.0 (28.4)	36.0 (26.5)	14.0 (50.0)	61.0 (26.9)	
Etiology, *n* (%)	190.0 (48.6)	66.0 (48.5)	23.0 (82.1)	101.0 (44.5)	
Clinical Presentation, *n* (%)	181.0 (46.3)	66.0 (48.5)	22.0 (78.6)	93.0 (41.0)	
Diagnosis, *n* (%)	262.0 (67.0)	86.0 (63.2)	25.0 (89.3)	151.0 (66.5)	
Treatment, *n* (%)	295.0 (75.4)	75.0 (55.1)	22.0 (78.6)	198.00 (87.2)	
Prevention, *n* (%)	61.0 (15.6)	12.0 (8.8)	4.0 (14.3)	45.0 (19.8)	
Prognosis, *n* (%)	155.0 (39.6)	59.0 (43.4)	8.0 (28.6)	88.0 (38.8)	
Video quality					
VIQI score, M (Q_1_, Q_3_)	13.0 (11.0, 14.0)	12.0 (11.0,13.0)	13.0 (12.8,14.0)	13.0 (12.0,14.0)	**<0**.**001**
GQS score, M (Q_1_, Q_3_)	3.0 (3.0, 4.0)	3.0 (2.0,4.0)	3.0 (3.0,4.0)	3.0 (3.0,4.0)	0.200
mDISCERN score, M (Q_1_, Q_3_)	2.0 (2.0, 2.0)	2.0 (2.0,2.0)	2.0 (2.0,2.0)	2.0 (2.0,2.0)	0.690
JAMA score, M (Q_1_, Q_3_)	2.0 (2.0, 2.0)	1.0 (1.0,2.0)	2.0 (2.0,2.0)	2.0 (2.0,2.0)	**<0**.**001**

M: Median, Q_1_: 1st Quartile, Q_3_: 3rd Quartile.

VIQI, Video Information Quality Instrument; GQS, Global Quality Score; mDISCERN, modified DISCERN tool; JAMA, JAMA Benchmarks. Data are presented as median (Q_1_, Q_3_). *P*-values were calculated using Kruskal–Wallis test. Bold values indicate statistical significance (*P* < 0.05).

Videos uploaded by individual users had the longest median duration [192.5 s (Q_1_, Q_3_: 76.8, 720.8)], followed by institutions [117.5 s (Q_1_, Q_3_: 83.8, 209.0)] and medical practitioners [83.0 s (Q_1_, Q_3_: 42.0, 203.5)] (*P* < 0.001). In contrast, videos uploaded by medical practitioners received the highest engagement, with a median of 278.0 likes, 53.0 collections, 30.0 comments, and 28.0 shares. Individual users had the lowest engagement across most indicators.

In terms of content coverage, videos uploaded by medical practitioners most frequently addressed treatment (198/227, 87.2%), diagnosis (151/227, 66.5%), and etiology (101/227, 44.5%). Institutional videos showed relatively broad coverage of diagnosis (25/28, 89.3%), etiology (23/28, 82.1%), clinical presentation (22/28, 78.6%), and treatment (22/28, 78.6%). Individual users more frequently covered prognosis than institutions and medical practitioners, but had lower coverage of treatment and prevention.

Regarding video quality, the VIQI score differed significantly among uploader categories (*P* < 0.001). Medical practitioners and institutions had higher VIQI scores than individual users. JAMA scores also differed significantly among uploader categories (*P* < 0.001), with individual users showing lower scores than medical practitioners and institutions. However, no significant differences were observed in GQS (*P* = 0.200) or mDISCERN scores (*P* = 0.690) among uploader categories. The distributions and comparisons of quality scores among uploader categories are shown in Figures 3B–E and Supplementary Figure S1, S2.

Further subgroup analysis was performed within each platform (Supplementary Table S1, Supplementary Table S2). On Bilibili, significant differences were found among uploader categories in video length, likes, comments, VIQI score, GQS score, and JAMA score. Individual users uploaded longer videos than medical practitioners and institutions. Institutions and medical practitioners generally achieved higher engagement than individual users. For quality assessment, institutions had the highest VIQI score, whereas individual users showed lower JAMA scores. No significant difference was observed in mDISCERN scores among uploader categories on Bilibili. On TikTok, video length did not differ significantly among uploader categories (*P* = 0.917). However, likes, collections, and shares differed significantly. Institutional videos received the highest median numbers of likes and shares, while medical practitioner videos showed higher collections than individual user videos. For quality assessment, medical practitioners had the highest VIQI score [14.0 (13.0, 14.0)], followed by institutions and individual users. GQS and JAMA scores also differed significantly among uploader categories, whereas mDISCERN scores did not differ significantly.

### 3.5 Multivariable analysis of factors associated with video quality

To determine whether platform differences remained independently associated with video quality after adjustment for potential confounding factors, multivariable regression analyses were performed with platform, uploader type, log-transformed video duration, and content coverage score included as covariates (Table 4 and Supplementary Tables S3–S5).

**Table 4:** Multivariable robust regression analysis of factors associated with VIQI scores.

Variables	*β* coefficient	95% CI	*P* value	FDR-adjusted *P*
Platform				
Bilibili (reference)	–	–	–	–
TikTok	0.819	0.512–1.127	**<0**.**001**	**<0**.**001**
Uploader type				
Individual users (reference)	–	–	–	–
Institutions	1.585	1.013–2.157	**<0**.**001**	**<0**.**001**
Medical practitioners	1.701	1.381–2.022	**<0**.**001**	**<0**.**001**
Video characteristics				
Log(video duration)	0.528	0.401–0.656	**<0**.**001**	**<0**.**001**
Content coverage score (0–7)	0.212	0.125–0.299	**<0**.**001**	**<0**.**001**

Model adjusted for platform, uploader type, log-transformed video duration, and content coverage score. β coefficients represent adjusted associations with VIQI score. VIQI, Video Information Quality Instrument; Bold values indicate statistical significance (*P* < 0.05).

In the robust regression model for VIQI scores, TikTok remained independently associated with higher VIQI scores compared with Bilibili (*β* = 0.819, 95% CI: 0.512–1.127, FDR-adjusted *P* < 0.001). Compared with individual users, videos uploaded by institutions (*β* = 1.585, 95% CI: 1.013–2.157, FDR-adjusted *P* < 0.001) and medical practitioners (*β* = 1.701, 95% CI: 1.381–2.022, FDR-adjusted *P* < 0.001) showed higher VIQI scores. Longer video duration and higher content coverage scores were also independently associated with higher VIQI scores.

In ordinal logistic regression analyses, medical practitioner-generated videos were associated with higher odds of achieving higher GQS scores (OR = 4.041, FDR-adjusted *P* < 0.001). Platform was not independently associated with GQS, mDISCERN, or JAMA scores after adjustment (Supplementary Tables S3–S5).

### 3.6 Spearman correlation analysis

Spearman correlation analysis was performed to explore the associations among video length, engagement indicators, and quality assessment scores (Figure 4). Strong positive correlations were observed among engagement indicators, including likes, collections, comments, and shares. For example, likes were strongly correlated with comments and shares, and collections were strongly correlated with shares, indicating that videos with high audience engagement tended to perform consistently across multiple interaction indicators.

**Figure 4:**
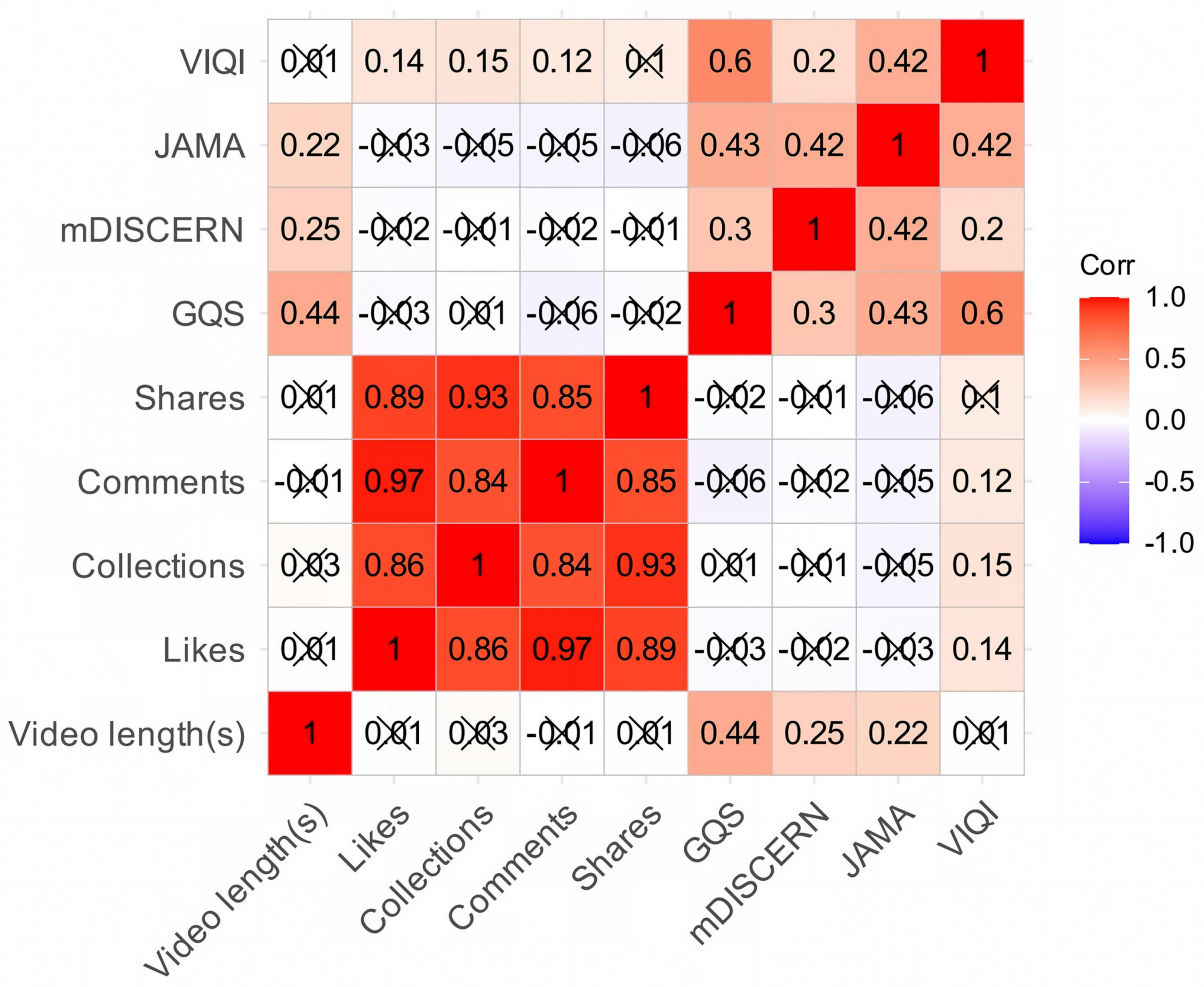
Spearman correlation matrix among video variables, engagement indicators, quality, and reliability scores. X: *P* ≥ 0.05.

Moderate positive correlations were observed among several quality assessment tools. GQS was positively correlated with VIQI, JAMA, and mDISCERN scores, while JAMA was also positively correlated with VIQI and mDISCERN scores. However, correlations between engagement indicators and quality scores were weak or not statistically significant. These findings suggest that video popularity did not necessarily reflect higher information quality or reliability.

## 4 Discussion

### 4.1 Principal findings

This study provides a comprehensive evaluation of TAAD-related short-video content on two major Chinese platforms, TikTok and Bilibili, focusing on engagement patterns, uploader characteristics, informational quality, and thematic coverage. Overall, short-video platforms showed considerable potential for disseminating TAAD-related health information; however, the reliability and completeness of the information remained limited. Among the 391 included videos, the overall video quality was moderate, with a median GQS score of 3.0 and a median VIQI score of 13.0. Nevertheless, the median mDISCERN score was only 2.0, suggesting insufficient reliability and limited use of evidence-based references. Engagement indicators were strongly interrelated but showed weak associations with validated quality or reliability scores, indicating that highly visible videos did not necessarily demonstrate higher information quality, reliability, or completeness. This discrepancy was particularly evident on TikTok, where likes, comments, and shares were significantly higher than on Bilibili despite comparable median reliability scores. Similar patterns have been reported in studies of other medical conditions on short-video platforms, where popularity metrics were not consistently associated with higher information quality or reliability (12, 19, 20). Although several comparisons reached statistical significance, some differences showed limited practical magnitude, particularly when median values and interquartile ranges were similar between groups. Therefore, statistical significance should be interpreted together with the magnitude of observed differences and practical relevance. Given the life-threatening nature of TAAD and the importance of timely recognition and follow-up care, these information gaps may have implications for public understanding and health-related decision-making (21, 22). Collectively, our findings highlight both the potential value and the limitations of short-form video platforms for public health education.

### 4.2 Platform differences and uploader characteristics

Content focus varied between platforms. Bilibili videos were significantly longer and showed higher coverage of epidemiology, etiology, and clinical presentation-related content, suggesting broader educational depth in selected disease-related domains. In contrast, TikTok videos were shorter but showed substantially higher audience engagement, including more likes, comments, and shares. TikTok videos showed higher VIQI, GQS, and mDISCERN scores in unadjusted comparisons, although median GQS and mDISCERN values were identical across platforms and JAMA scores did not differ significantly. The higher proportion of medical professionals on TikTok may partly explain these differences (23). However, concerns regarding the quality and reliability of health information on short-video platforms remain, as lower-quality or incomplete health information may be widely disseminated (24, 25). Therefore, platform differences should be interpreted cautiously rather than as evidence of overall superiority of one platform.

Uploader composition may partly explain these differences. Medical practitioners accounted for 74.7% of TikTok videos but only 40.9% of Bilibili videos, whereas individual users were the largest uploader group on Bilibili. Overall, medical practitioners contributed the largest proportion of videos and achieved higher engagement than individual users. They also showed better VIQI and JAMA scores, indicating more structured information presentation and greater transparency. Videos produced by medical professionals or institutions generally achieved higher scores in information presentation and source disclosure, suggesting that professional participation may improve some aspects of health communication (20, 26, 27). However, mDISCERN scores remained comparable across uploader categories, indicating that professional identity alone does not guarantee higher reliability or completeness of health information. In addition, professional content did not consistently achieve greater engagement or visibility, highlighting that information quality and audience engagement may be influenced by different factors. In contrast, non-professional videos showed less complete coverage of several assessed content domains and lower scores on some quality measures. Similar observations have been reported in a recent cross-sectional study of Wilson disease-related videos across Bilibili, Douyin, and Kuaishou, which highlighted the roles of platform characteristics and uploader identity in medical video quality (28). This finding echoes previous studies indicating social media ecosystems are characterized by heterogeneous contributors, where professional authority does not always translate into greater visibility (29–31). Furthermore, content coverage score was independently associated with several quality measures, suggesting that the comprehensiveness of disease-related information may be an important determinant of assessed video quality beyond platform and uploader characteristics.

The association between uploader type and video quality appeared to vary across platforms. On TikTok, medical practitioners and institutions generally achieved higher VIQI, GQS, and JAMA scores than individual users, consistent with previous evidence that professionally generated health videos tend to show better overall quality (16, 32). On Bilibili, the pattern was less consistent, possibly reflecting its longer and more structured educational format, which may improve perceived usefulness without necessarily enhancing evidence-based reliability (32). Notably, mDISCERN scores did not differ among uploader types on either platform, similar to findings in knee arthroplasty videos (32). These findings suggest that professional identity alone may not ensure reliable content, highlighting the need for clearer evidence citation and source disclosure.

Together, these findings indicate that improving the quality of TAAD-related short videos requires not only greater professional participation but also platform-level mechanisms that better align content visibility with informational reliability.

### 4.3 Correlation analysis

Spearman correlation analysis provided further insight into the dissemination dynamics of TAAD-related short videos. Strong positive correlations were observed among likes, collections, comments, and shares, suggesting that engagement indicators are highly co-dependent. This pattern may reflect platform recommendation mechanisms, in which videos receiving early audience interaction are more likely to be further promoted. In contrast, video length showed no meaningful correlation with engagement indicators. Although TikTok videos were generally shorter and received higher engagement at the platform level, duration itself did not appear to be the sole determinant of popularity. Moderate positive correlations were observed among several quality assessment tools, indicating that GQS, mDISCERN, JAMA, and VIQI capture related but distinct dimensions of video quality. Most importantly, engagement metrics were not strongly correlated with the assessed quality or reliability scores. These findings indicate that viral popularity did not correspond closely with the quality and reliability measures used in this study. This pattern has been documented in studies on other health topics, such as premature ovarian failure and thyroid eye disease, on similar platforms (33, 34). This analysis suggests that engagement metrics alone may be insufficient for evaluating the quality and reliability of health information online.

### 4.4 Content coverage gaps and clinical implications

A pronounced imbalance in thematic coverage was observed. Treatment was the most frequently addressed topic, appearing in 295 videos (75.4%), followed by diagnosis in 262 videos (67.0%). In contrast, prevention was covered in only 61 videos (15.6%), making it the least represented topic, while prognosis was discussed in 155 videos (39.6%). This emphasis on acute management, with comparatively limited coverage of prevention and long-term outcomes, indicates an imbalance in the information presented to viewers (21). The limited coverage of prognostic information is also notable. Limited discussion of prognosis may reduce the completeness of information available to patients and families facing this severe diagnosis (22). Incomplete coverage of the disease trajectory may limit the educational utility of widely consumed social media content. Future educational initiatives should prioritize balanced content portfolios that include evidence-based prevention strategies, realistic prognostic discussions, and psychosocial support resources.

### 4.5 Implications for practice

Driven by widespread internet access and a societal push for greater health literacy, patients are no longer passive recipients but have become active participants in seeking health information (35). Our findings have several important implications. First, healthcare professionals and cardiovascular societies should take a more active role in producing accessible, engaging, and evidence-based short-video content. Second, platforms should consider implementing reliability-enhancing mechanisms, such as verified medical uploader labels, standardized disclaimers, or partnerships with academic institutions. Such measures may help users identify content with clearer source attribution and professional accountability. Third, platforms could consider incorporating information-quality indicators or expert review mechanisms alongside existing content-selection strategies to support the dissemination of higher-quality health information. Finally, clinicians may consider integrating curated short-video resources into patient education strategies, using social media as an entry point while ensuring depth and continuity through formal healthcare systems. In a word, a collaborative effort is required to improve the digital health information ecosystem (36).

### 4.6 Strengths and limitations

This study provides a systematic evaluation of TAAD-related video content on two major Chinese short-video platforms: TikTok and Bilibili. TikTok attracts a broad, demographically diverse audience, while Bilibili primarily serves a younger user base. Studying both platforms thus provides a broader understanding of TAAD-related health information across different short-video environments, mitigating the biases inherent in single-platform analyses (13). The use of multiple validated assessment tools (GQS, mDISCERN, JAMA, VIQI) enabled a comprehensive, multidimensional analysis, while platform and uploader comparisons offer insights into factors associated with health information quality on social media.

Several limitations should be noted. The sample of 391 videos was relatively limited, which may affect the generalizability of the results. The cross-sectional design captures only a snapshot in time, whereas video content, engagement patterns, and platform ranking mechanisms may change dynamically over time (37). Although standardized search conditions were applied, residual effects of algorithm-dependent ranking and personalization cannot be completely excluded. The use of a single search term and restriction to top-ranked videos may also limit the representativeness of the sample. Differences in uploader composition between platforms may have influenced observed quality differences; although multivariable analyses were performed to adjust for platform, uploader type, video duration, and content coverage, residual confounding from unmeasured factors remains possible. Engagement metrics were analyzed using absolute counts of likes, comments, collections, and shares, which may be affected by differences in video exposure time. Therefore, these comparisons should be interpreted cautiously. Multiple statistical comparisons were performed, and although FDR correction was applied for multivariable models and content coverage comparisons, other comparative and correlation analyses were considered exploratory. In addition, the content assessment focused on broad TAAD-related domains and did not include a disease-specific checklist evaluating time-critical information, such as symptom recognition, emergency care, and timely referral. Finally, the present study evaluated information quality, reliability, completeness, and transparency using established assessment instruments but did not independently verify individual medical statements against current TAAD guidelines or other authoritative clinical evidence. Therefore, the findings should not be interpreted as a direct assessment of factual accuracy or misinformation.

## 5 Conclusions

This study provides a systematic evaluation of TAAD-related short videos on Chinese social media platforms, revealing overall suboptimal informational quality characterized by acceptable production standards but limited reliability. Although TikTok showed higher user engagement and remained independently associated with higher VIQI scores after adjustment, uploader characteristics and content coverage were also important determinants of assessed information quality. Engagement metrics showed weak or no associations with validated quality and reliability scores, suggesting that popularity alone may not reflect the assessed information quality of TAAD-related videos. This gap, together with limited coverage of preventive and prognostic information, may have implications for public understanding and health-related decision-making, particularly given the time-sensitive nature of TAAD. These findings support the need for coordinated efforts to improve the quality, reliability, completeness, and transparency of TAAD-related health information on short-video platforms. From a platform perspective, measures such as clearer source disclosure, professional verification, and information-quality review mechanisms may help improve the transparency and reliability of health-related content. At the individual level, viewers should be encouraged to critically appraise short-video content, consider source attribution and professional credentials, and avoid relying on engagement metrics alone when evaluating health information.

## Data Availability

The datasets presented in this study can be found in online repositories. The names of the repository/repositories and accession number(s) can be found in the article/Supplementary Material.
