# Supplementary material for: Quality and reliability of short videos about type A aortic dissection on Bilibili and TikTok: a cross-sectional study
Source: Front Digit Health. 2026 Oct 21;8:1964412. doi: 10.3389/fdgth.2026.1964412 (PMC13732769; doi:10.3389/fdgth.2026.1964412)
Supplement: Supplementary file 1 [file Supplementaryfile1.docx]

**Table S 1** Characteristics, quality, and reliability of TAAD videos by different uploaders on TikTok.

| Variables | Total  (n = 198) | Individual Users  (n = 40) | Institutions  (n = 10) | Medical practitioners  (n = 148) | *P* |
| --- | --- | --- | --- | --- | --- |
|  |  |  |  |  |  |
| **General information** |  |  |  |  |  |
| Video length(s), M (Q₁, Q₃) | 77.0 (40.0, 143.0) | 73.0 (32.5,127.0) | 92.5 (42.8,121.0) | 77.0 (41.5,145.5) | 0.917 |
| Likes, M (Q₁, Q₃) | 245.5 (95.5, 812.5) | 104.0 (57.0,159.0) | 765.5 (40.5,2648.8) | 329.0 (127.3,997.8) | **<.001** |
| Collections, M (Q₁, Q₃) | 37.5 (6.3, 215.0) | 5.0 (3.0,18.0) | 56.5 (4.5,283.3) | 52.5 (13.8,234.8) | **<.001** |
| Comments, M (Q₁, Q₃) | 51.0 (13.0, 180.8) | 57.0 (20.8,165.0) | 85.5 (2.0,205.8) | 45.0 (12.8,170.3) | 0.714 |
| Shares, M (Q₁, Q₃) | 24.00 (5.0, 160.3) | 5.5 (2.0,17.0) | 187.0 (8.3,409.8) | 36.0 (7.0,167.3) | **<.001** |
| **Video content** |  |  |  |  |  |
| Epidemiology,n(%) | 44.0 (22.2) | 2.0 (5.0) | 4.0 (40.0) | 38.0 (25.7) |  |
| Etiology,n(%) | 79.0 (39.9) | 7.0 (17.5) | 8.0 (80.0) | 64.0 (43.2) |  |
| Clinical Presentation,n(%) | 73.0 (36.9) | 7.0 (17.5) | 9.0 (90.0) | 57.0 (38.5) |  |
| Diagnosis,n(%) | 123.0 (62.1) | 12.0 (30.0) | 10.0 (100.0) | 101.0 (68.2) |  |
| Treatment,n(%) | 152.0 (76.8) | 14.0 (35.0) | 9.0 (90.0) | 129.0 (87.2) |  |
| Prevention,n(%) | 33.0 (16.7) | 3.0 (7.5) | 3.0 (30.0) | 27.0 (18.2) |  |
| Prognosis,n(%) | 89.0 (44.9) | 34.0 (85.0) | 3.0 (30.0) | 52.0 (35.1) |  |
| **Video quality** |  |  |  |  |  |
| VIQI score, M (Q₁, Q₃) | 13.0 (12.0, 14.0) | 11.0 (10.0,11.3) | 13.0 (13.0,14.0) | 14.0 (13.0,14.0) | **<.001** |
| GQS score, M (Q₁, Q₃) | 3.0 (3.0, 4.0) | 2.0 (2.0,2.0) | 3.0 (3.0,3.0) | 3.0 (3.0,4.0) | **<.001** |
| mDISCERN score, M (Q₁, Q₃) | 2.0 (2.0, 2.0) | 2.0 (2.0,2.0) | 2.0 (2.0,2.0) | 2.0 (2.0,2.0) | 0.504 |
| JAMA score, M (Q₁, Q₃) | 2.0 (2.0, 2.0) | 1.0 (1.0,1.0) | 2.0 (2.0,2.0) | 2.0 (2.0,2.0) | **<.001** |
| M: Median, Q₁: 1st Quartile, Q₃: 3st Quartile  VIQI:Video Information Quality Instrument;GQS: Global Quality Score; mDISCERN: modified DISCERN tool;JAMA:JAMA Benchmarks.Data are presented as median (Q1, Q3). P-values were calculated using Kruskal-Wallis test. Bold values indicate statistical significance (P < 0.05) | | | | | |

**Table S 2** Characteristics, quality, and reliability of TAAD videos by different uploaders on Bilibili.

| Variables | Total  (n = 193) | Individual Users  (n = 96) | Institutions  (n = 18) | Medical practitioners  (n = 79) | *P* |
| --- | --- | --- | --- | --- | --- |
|  |  |  |  |  |  |
| **General information** |  |  |  |  |  |
| Video length(s), M (Q₁, Q₃) | 213.0 (86.0, 668.0) | 291.5 (96.0,908.5) | 155.5 (107.8,476.5) | 147.0 (42.5,275.5) | **<.001** |
| Likes, M (Q₁, Q₃) | 33.0 (7.0, 303.0) | 24.0 (6.8,91.0) | 93.5 (30.5,237.3) | 90.0 (6.0,716.5) | **0.013** |
| Collections, M (Q₁, Q₃) | 49.0 (6.0, 179.0) | 41.0 (10.5,144.3) | 68.0 (31.8,205.0) | 53.0 (3.0,200.5) | 0.386 |
| Comments, M (Q₁, Q₃) | 4.0 (0.0, 38.0) | 2.0 (0.0,7.3) | 8.0 (1.3,22.5) | 7.0 (1.0,65.0) | **0.007** |
| Shares, M (Q₁, Q₃) | 14.0 (2.0, 59.0) | 13.0 (2.0,52.3) | 14.5 (7.5,91.0) | 16.0 (0.5,67.5) | 0.428 |
| **Video content** |  |  |  |  |  |
| Epidemiology,n(%) | 67.0 (34.7) | 34.0 (35.4) | 10.0 (55.6) | 23.0 (29.1) |  |
| Etiology,n(%) | 111.0 (57.5) | 59.0 (61.5) | 15.0 (83.3) | 37.0 (46.8) |  |
| Clinical Presentation,n(%) | 108.0 (56.0) | 59.0 (61.5) | 13.0 (72.2) | 36.0 (45.6) |  |
| Diagnosis,n(%) | 139.0 (72.0) | 74.0 (77.1) | 15.0 (83.3) | 50.0 (63.3) |  |
| Treatment,n(%) | 143.0 (74.1) | 61.0 (63.5) | 13.0 (72.2) | 69.0 (87.3) |  |
| Prevention,n(%) | 28.0 (14.5) | 9.0 (9.4) | 1.0 (5.6) | 18.0 (22.8) |  |
| Prognosis,n(%) | 66.0 (34.2) | 25.0 (26.0) | 5.0 (27.8) | 36.0 (45.6) |  |
| **Video quality** |  |  |  |  |  |
| VIQI score, M (Q₁, Q₃) | 12.0 (11.0, 14.0) | 12.0 (11.0,13.0) | 13.0 (12.0,14.8) | 13.0 (11.0,14.0) | **0.007** |
| GQS score, M (Q₁, Q₃) | 3.0 (3.0, 4.0) | 4.0 (3.0,4.0) | 4.0 (3.0,4.0) | 3.0 (3.0,4.0) | **0.028** |
| mDISCERN score, M (Q₁, Q₃) | 2.0 (2.0, 2.0) | 2.0 (2.0,2.0) | 2.0 (2.0,2.0) | 2.0 (2.0,2.0) | 0.846 |
| JAMA score, M (Q₁, Q₃) | 2.0 (2.0, 2.0) | 2.0 (1.0,2.0) | 2.0 (2.0,2.0) | 2.0 (2.0,2.0) | **<.001** |
| M: Median, Q₁: 1st Quartile, Q₃: 3st Quartile  VIQI:Video Information Quality Instrument;GQS: Global Quality Score; mDISCERN: modified DISCERN tool;JAMA:JAMA Benchmarks.Data are presented as median (Q1, Q3). P-values were calculated using Kruskal-Wallis test. Bold values indicate statistical significance (P < 0.05). | | | | | |

**Table S 3** Ordinal logistic regression analysis of factors associated with GQS scores.

| Variables | OR | 95% CI | *P* value | FDR-adjusted *P* |
| --- | --- | --- | --- | --- |
|  |  |  |  |  |
| **Platform** |  |  |  |  |
| Bilibili (reference) | - | - | - | - |
| TikTok | 0.638 | 0.397-1.023 | 0.062 | 0.078 |
| **Uploader type** |  |  |  |  |
| Individual users (reference) | - | - | - | - |
| Institutions | 2.087 | 0.870-5.010 | 0.100 | 0.100 |
| Medical practitioners | 4.041 | 2.402-6.795 | **<0.001** | **<0.001** |
| **Video characteristics** |  |  |  |  |
| Log(video duration) | 3.000 | 2.371-3.797 | **<0.001** | **<0.001** |
| Content coverage score (0-7) | 1.691 | 1.455-1.965 | **<0.001** | **<0.001** |
| ORs represent adjusted odds ratios from ordinal logistic regression models. GQS: Global Quality Score; Bold values indicate statistical significance (*P* < 0.05). | | | | |

**Table S 4** Ordinal logistic regression analysis of factors associated with mDISCERN scores.

| Variables | OR | 95% CI | *P* value | FDR-adjusted *P* |
| --- | --- | --- | --- | --- |
|  |  |  |  |  |
| **Platform** |  |  |  |  |
| Bilibili (reference) | - | - | - | - |
| TikTok | 0.978 | 0.401-2.388 | 0.962 | 0.962 |
| **Uploader type** |  |  |  |  |
| Individual users (reference) | - | - | - | - |
| Institutions | 0.733 | 0.160-3.356 | 0.689 | 0.862 |
| Medical practitioners | 1.565 | 0.647-3.783 | 0.320 | 0.534 |
| **Video characteristics** |  |  |  |  |
| Log(video duration) | 2.036 | 1.426-2.906 | **<0.001** | **<0.001** |
| Content coverage score (0-7) | 1.229 | 0.975-1.547 | 0.080 | 0.200 |
| ORs represent adjusted odds ratios from ordinal logistic regression models. mDISCERN: modified DISCERN tool; Bold values indicate statistical significance (*P* < 0.05). | | | | |

**Table S 5** Ordinal logistic regression analysis of factors associated with JAMA scores.

| Variables | OR | 95% CI | *P* value | FDR-adjusted *P* |
| --- | --- | --- | --- | --- |
|  |  |  |  |  |
| **Platform** |  |  |  |  |
| Bilibili (reference) | - | - | - | - |
| TikTok | 0.717 | 0.351-1.464 | 0.361 | 0.361 |
| **Uploader type** |  |  |  |  |
| Individual users (reference) | - | - | - | - |
| Institutions | 69.079 | 16.881-282.570 | **<0.001** | **<0.001** |
| Medical practitioners | 149.759 | 55.408-404.704 | **<0.001** | **<0.001** |
| **Video characteristics** |  |  |  |  |
| Log(video duration) | 2.273 | 1.675-3.085 | **<0.001** | **<0.001** |
| Content coverage score (0-7) | 1.287 | 1.075-1.530 | 0.006 | 0.007 |
| ORs represent adjusted odds ratios from ordinal logistic regression models. JAMA:JAMA Benchmarks; Bold values indicate statistical significance (*P* < 0.05). | | | | |

**Figure S 1** Distribution and comparison of video quality scores among different uploader types.

A-D: Distribution of GQS/mDISCERN/JAMA/VIQI scores among different uploader types;

E-H: Comparison of GQS/mDISCERN/JAMA/VIQI scores among medical practitioners, individual users, and institutions.

(ns: P ≥ 0.05; ***P < 0.001; ****P < 0.0001)

**Figure S 2** Comparison of video quality scores among different uploader types on Bilibili and TikTok.

A-C: Comparison of mDISCERN/JAMA/VIQI scores among different uploader types on Bilibili;

D-F: Comparison of mDISCERN/JAMA/VIQI scores among different uploader types on TikTok.

(ns: P ≥ 0.05; *P < 0.05; ***P < 0.001; ****P < 0.0001)
